# Long-distance caregiving at the end of life: a protocol for an exploratory qualitative study in Germany

**DOI:** 10.1186/s12904-022-00967-8

**Published:** 2022-05-12

**Authors:** Franziska A. Herbst, Nils Schneider, Stephanie Stiel

**Affiliations:** Institute for General Practice and Palliative Care, Hannover Medical School, Carl-Neuberg-Straße 1, 30625 Hannover, Germany

**Keywords:** Palliative care, End-of-life care, Long-distance caregiving, Family caregivers, Adults, Family support, Social support, Interpersonal relations, Burden, Germany

## Abstract

**Background:**

Of the approximately 4.7 million people in Germany caring for a relative, many live at a geographical distance from their loved one. The provision of remote care to a terminally ill patient is associated with specific challenges and burdens. In the German context, research is lacking on the specific experiences and needs of caregivers in end-of-life situations who are geographically distanced from their relative. Thus, the overarching goal of the proposed study is to detail the specifics of long-distance caregiving at the end of life in Germany, determining the role played by physical distance in shaping end-of-life caregiving and identifying the needs of long-distance caregivers in this situation.

**Methods:**

The exploratory qualitative study will be guided by an inductive logic, drawing on one-time semi-structured interviews. To uncover the multiplicity of caregiving experiences, long-distance caregivers of both patients receiving early palliative care and patients at a very advanced stage of disease will be included. The study will be divided into five phases: (1) preparation and pretest, (2) data collection and primary analysis, (3) data analysis and interpretation, (4) advisory board workshop and (5) conclusions and recommendations.

**Discussion:**

The study will aim at generating valuable insight regarding the experiences and needs of family caregivers of end-of-life patients. This is particularly relevant, given that families are becoming increasingly geographically dispersed. As this trend continues, it will challenge traditional models of family care and shed light on novel caregiving issues that will need to be addressed through social and health policy.

**Trial registration:**

The study was prospectively registered in the German Clinical Trials Register (Deutsches Register Klinischer Studien) (Registration N° DRKS00024164; date of registration: January 25, 2021), and is searchable under the International Clinical Trials Registry Platform Search Portal of the World Health Organization, under the German Clinical Trials Register number.

**Supplementary Information:**

The online version contains supplementary material available at 10.1186/s12904-022-00967-8.

## Background

### Problem area: demographic trends and long-distance caregiving

In Germany, there are more than 3.41 million individuals in need of long-term care. Three-quarters (2.59 million) of these individuals are cared for at home, and 1.76 million of them are cared for by relatives, alone [1]. Currently, approximately 4.7 million family caregivers in Germany are providing care for a relative [2]. Against this backdrop, the provision of care is becoming complicated by socio-demographic trends, showing that an increasing number of adult children are living at a distance from their aging parents and siblings, having relocated to attend university, pursue a job, accompany a partner or experience another country and culture [3–5]. Moreover, individuals with an immigrant background, who represent 23.6% of Germany’s [6] population, are likely to have family living abroad. Given these shifting lifestyle factors and demographics—driven by migration, globalization and population change [7–10]—it is not surprising that long-distance caregiving (LDC) has become a common practice across the globe. In addition, the COVID-19 pandemic, and its associated travel restrictions and social distancing requirements, is hindering caregivers from visiting their loved ones to provide local support. However, long-distance (LD) caregivers can still provide significant support to kin by assuming the role of care manager and coordinating daily routines from afar [11].

### Conceptual definition: long-distance caregiving

In the literature, the phenomenon of caring for a relative from a distance has been referred to as “distance caregiving” or “long-distance caregiving” (LDC). Casañas i Comabella [12] notes that there is no consensus on an operational definition for LDC. Some studies use mileage [13, 14] to define distance, while others base their definition on travelling time [15]. Bledsoe et al. [16] operationalize LDC as an effort made by family members who “reside at a location that is sufficiently geographically distant that the caregiver cannot have daily face-to-face contact with the relative.” More recent research emphasizes subjective conceptualizations of distance, allowing family caregivers and caregiving recipients to determine for themselves whether they are in an LDC situation. This idea is based on the assumption that relational partners are experts on their own situation and the challenges they face (which may even arise in situations of minimal distance) [3, 12]. However, all definitions share a conception of the LD caregiver as “a family carer who is restricted in how often they can visit because they do not live nearby” [12] and who perceives communication opportunities to be restricted by distance [3].

### Current state of research

Only a handful of international studies are available on LDC in end-of-life contexts [10, 12, 17–20]. In Germany, research on the topic of LDC, in general, has only recently been initiated [21], and research on the specific experiences and needs of LD caregivers in end-of-life contexts is lacking. However, research on remote caregiving in end-of-life contexts is of particular importance, because the costs of caring for a terminally ill patient differs from the costs of general LDC.

In general, the challenges experienced by LD caregivers may be exacerbated by work commitments, with serious implications for caregivers’ health, social isolation and finances [3, 7, 9, 10, 22, 23]. For instance, it is well documented that cancer and other life-threatening illnesses tend to severely affect the health and well-being of family members [24–27]. Family caregivers may also experience stress when breaking bad news to other family members, and they may suffer mental health problems if they are unprepared for the death of their loved one [22, 23, 28]. Although LD caregivers experience burdens similar to those expressed by local caregivers, LD caregivers describe additional burdens unique to their LDC situation, underscoring the particular difficulties of managing distance in this context [3]. Furthermore, LD caregivers experience emotional suffering and report feelings of sadness, helplessness, anger, grief, frustration, anxiety and guilt [3, 12, 20, 29, 30], due to their geographical distance from their loved one at the end of life, which prevents them from getting to their relative quickly. Indeed, they often worry about their ability to make a timely arrival in the event of a crisis [20]. LD caregivers may express a wish to do more for their family member [3, 15, 31], experience uncertainty about the ideal visiting time and struggle with the social expectation that close kin—particularly children—are expected to visit end-of-life patients in person [11]. Moreover, LD caregivers may struggle to cover travel costs and take extended leave from work without pay [11]. Refugees face the additional obstacle of being unable to leave the country due to restrictions linked to their immigration status [11]. Finally, given their reliance on second-hand information, LD caregivers often express a need for more information about their loved one’s disease, therapy and prognosis [20]. However, LD caregivers also describe positive aspects of caring from a distance, such as an appreciation of their ability to contribute to their relative’s care [29]. In Mazanec’s [20] study, adult child caregivers experienced an intensification of their relationship with their ill parent, due to their numerous phone conversations. Finally, LD caregivers express a perceived benefit of not being exposed to the terminal illness experience every day, but being able to step away and engage in leisure activities [12, 20].

### Research gaps and open questions

Bevan et al. call for further study on aspects of LDC communication, as the topic is largely unexplored and further research could minimize the challenges experienced by LD caregivers [3]. There is also a lack of research on interpersonal conflict management in the context of LDC relationships [3, 32], extending to tensions between LD caregivers, their spouses and their children, as well as to conflicts with local caregiving siblings [29, 33, 34].

Cagle and Munn [7], in their systematic review of the LDC literature, call for further research on diverse—particularly vulnerable—populations. Gendered expectations of caregiving frequently challenge women, who may be forced down a tightrope if confronted with managing a career, raising children and caregiving for a relative. In this context, conflict, distress and career sacrifices for female caregivers should be foregrounded, given evidence that distance may aggravate these negative consequences for female caregivers by preventing them from meeting their caregiving self-expectations [7, 35, 36]. Further research is also needed on cultural factors, which affect caregiving experiences, expectations and relationships—particularly within the family [36–40].

## Study aims

The project will explicitly address caregivers, as these figures often put aside their own needs to focus on providing support for a loved one [41]. The overarching goal of the project will be to detail the specifics of LDC at the end of life in Germany, outlining the ways in which distance shapes end-of-life caregiving and identifying the needs of end-of-life LD caregivers. More specifically, the project will seek to uncover caregivers’ perceptions and experiences of LDC, and how these might vary amongst caregivers from different cultural backgrounds. Ideas of what “good care” means at the end of life will also be explored, considering relevant aspects of diversity.

The two core research questions will be: (1) How do caregivers experience LDC for a relative at the end of life (in terms of, e.g., the caregiver’s life, health, and well-being; emotional, physical and financial burdens; and interpersonal conflict with the care recipient and/or other family members)? and (2) What are the specific (support) needs of end-of-life LD caregivers and what support do they desire and need, both for themselves and for the care recipient? Accordingly, the project will: (i) describe the specifics of LDC at the end of life and (ii) recommend support interventions for LD caregivers.

This exploratory research project will generate and disseminate scientific knowledge on LDC based on LD caregivers’ own experiences, understandings and perceived needs, and thereby fill knowledge gaps in the literature and contribute to opening new directions for LD caregiver research. Furthermore, the project will aim at producing knowledge to inform society and policy makers, at both local and national levels, regarding support for family caregivers of end-of-life patients. Such knowledge is critically needed, given the increasing number of individuals living at a geographical distance from their family. As this trend intensifies, traditional models of care within families will be challenged, shedding light on novel caregiving issues that will need to be addressed through social and health policy.

### Gender and diversity issues

Torensma et al.’s [42] self-assessment instrument, “Diversity Responsiveness in Palliative Care Projects,” will be administered throughout the project to ensure responsiveness to diversity issues. Furthermore, the data analysis will attend to the following diversity questions: (1) What roles do culture, gender and other issues of diversity play in end-of-life LDC? (2) How are space and spatial distance experienced differently by caregivers from different cultural backgrounds? and (3) How do caregivers experience LDC differently when their loved one lives abroad (cf. Zechner 2007 [5])? The project will use interpreters to facilitate the inclusion of neglected groups, such as foreign caregivers and vulnerable populations (e.g. caregivers with an immigrant background). Finally, the data will capture caregiver/caregiving characteristics pertaining to kin relationships, cultural background, place of residency and duration of the LDC relationship.

## Methods and design

The exploratory qualitative study will be guided by an inductive logic, drawing on one-time semi-structured interviews [43–45]. This qualitative exploratory approach is considered most appropriate for exploring the understudied phenomenon of LDC at the end of life, as it can contribute to describing LD caregivers’ perceptions and experiences of caregiving. As Stenberg et al. [46] note, few studies have addressed variations in caregiving responsibilities and challenges between different phases of a disease trajectory. However, it is known that psychological burden typically predominates in the early stage of cancer treatment, whereas psychosocial and physical problems increase in later stages. In addition, LD caregivers are likely to, over time, develop strategies to manage physical distance. To better understand such caregiving experiences, the study will include LD caregivers of both patients receiving early palliative care and patients in a very advanced stage of disease. The one-time interview approach with LD caregivers of patients across this spectrum will allow the research to cover a variety of caregiving experiences in a short amount of time and with a relatively low burden on individual caregivers, representing an advantage over longitudinal designs. Ensuring the long-term participation of end-of-life caregivers over a substantial period of time would be difficult, and dropout rates (due to withdrawal or the death of the patient) would likely be high [47–50].

Socio-demographic data will also be captured to provide background information on the participating LD caregivers. The study protocol will adhere to STROBE guidelines [51].

**Fig. 1:**
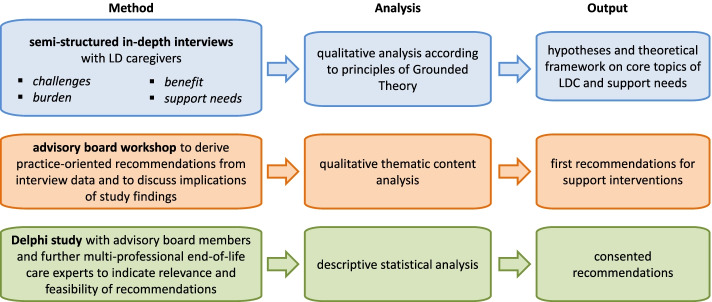
Study design

The project will be divided into five phases, spanning 2 years: (1) preparation and pretest (project months 1–4), (2) data collection and primary analysis (project months 5–16), (3) data analysis and interpretation (project months 17–19), (4) advisory board workshop (project months 20–21) and (5) conclusions and recommendations (project months 22–24). Figure 1 presents an overview of the study design.

### Work schedule, time frame and milestones

#### Phase 1: preparation and pretest

In study phase 1, the principal investigator will establish the infrastructure for the advisory board and study sample via: (1) established contacts with general practitioners, palliative care units, hospices and specialized palliative home care teams and (2) public relations. A systematic literature search will be conducted at the beginning of the project to understand the current state of research, guided by the following questions: (1) What does the existing literature tell us about LD caregiving at the end of life? and (2) What is known about the support needs of end-of-life LD caregivers? Literature from the fields of medicine, psychology, health services and related disciplines will be reviewed, including contributions published in English and German.

Study materials will also be prepared in this phase. In particular, the interview guide and a quantitative assessment instrument for family caregivers in end-of-life situations will be developed on the basis of the literature review for this study [52, 53]. Both the guide and the instrument will be discussed and scrutinized in a half-day advisory board meeting and a pretest will be conducted with two to three LD caregivers, to ensure the comprehensibility and appropriateness of the questions and length. The interview guide developed for this study is provided as Additional file 1. Moreover, the advisory board will review the project design and methodology, including the specifics of the recruitment strategy via general practitioners, palliative care units, hospices, specialized palliative home care teams and public relations, as well as the involvement of interpreters.

#### Phase 2: data collection and primary analysis

In study phase 2, the individual interviews with LD caregivers, including the contextualizing socio-demographic assessments, will be administered. In more detail, one of the two researchers will present the research project to eligible LD caregivers and invite them to participate in an individual semi-structured in-depth interview. Research methods guidelines hold that, in most projects, at least 12 interviews are required to achieve data saturation [54]. In the proposed project, a greater number of interviews will be needed, because the project will apply purposeful sampling to include participants with heterogeneous characteristics, with regards to gender, age, immigrant background, employment status and urban/rural residency. Moreover, the study will consider a similar range of characteristics amongst care recipients, on the assumption that such features may reflect meaningful differences in experiences and needs [55, 56]. For instance, the project will include patients of varying ages and kinship relations to the LD caregiver, in order to examine any effects of these variables [55, 56]. In total, the project will enroll approximately 30–35 LD caregivers. Sample size was approximated based on “information power” related to study aim, sample specificity, theoretical background, quality of the interview dialogue, and the analysis strategy [57]. Throughout the interview process, the data will be continuously analyzed to determine when data saturation is achieved; at this point, data collection will be terminated [47–50]. All participating LD caregivers whose mother tongue is not German will be offered an interpreter.

The interviews will focus on the impact of distance on end-of-life caregiving, LD caregivers’ perceptions and experiences of LDC and LD caregivers’ wishes and support needs with respect to the caregiving situation, with a particular focus on caregivers’ perceptions of the challenges, burdens and benefits of LDC. Participants will also be asked to provide socio-demographic data (i.e. age, gender, ethnicity, employment, relationship status, living arrangements). Following each qualitative interview, supplementary quantitative data will be collected on the frequency and duration of contact in the caregiving relationship and the nature of the LD caregiving support.

All qualitative interviews will be digitally recorded and transcribed verbatim by the research assistant, using the transcription software f4 (dr. dresing & pehl GmbH, Marburg, Germany). Each interview transcript will be analyzed by two researchers, using the qualitative data analysis software MAXQDA (VERBI GmbH, Berlin, Germany), based on grounded theory [58, 59]. Grounded theory, characterized by a bottom-up analysis, is considered effective for providing insight into largely unexplored social phenomena. Integrating case and categorical perspectives, it aims at producing a systematic understanding of peoples’ attitudes, experiences and expectations [60]. The method draws on the principle of induction, whereby theoretical statements are developed from and checked against the data. In this way, new theories are developed, grounded in the data.

In this study phase, two researchers will independently code the interview transcripts, using an inductive process. Codes with similar content will be grouped under concepts representing core topics. Coding will occur iteratively, with each interview coded shortly after the interview takes place.

In a second half-day advisory board meeting in project month 8, the preliminary findings of the first three months (months 5–7) of interviewing will be discussed. Interviews and participant recruitment will be reflected upon, with respect to both content and method. If necessary, the interview guide and recruitment strategy will be modified. All socio-demographic questionnaire data will be entered into IBM SPSS Statistics (SPSS Inc., Chicago, IL, USA).

#### Phase 3: data analysis and interpretation

In study phase 3, the researchers will relate similar concepts into broader categories, using an axial coding strategy. Throughout the coding process, theoretical memos will be written to define codes, concepts and categories and to trace the process of idea development. Based on the resulting codes, concepts and categories, hypotheses on the addressed social phenomena will be built. Socio-demographic data will be analyzed using descriptive statistics, and this contextualizing data will be drawn on to characterize participants. Interview data will be used to develop the hypotheses and consolidated into a conceptual framework of core LDC topics and support needs (e.g. psychosocial, information- and communication-related, instrumental and financial).

Throughout the data analysis, data bias will be openly addressed. The evaluation of qualitative data will be contextually bound, considering participants’ origins, values, norms, interests and motivations, among other factors. The heterogeneity of the participants and their different points of view, which the qualitative interviews will aim at capturing, are highly relevant to the research goal of capturing subjective perspectives on LDC and integrating them into a more holistic picture.

#### Phase 4: advisory board workshop

After the collected material from study phase 2 is integrated in study phase 3, the conceptual framework will be presented and discussed in a half-day advisory board workshop involving experts from end-of-life care research and practice. The workshop will aim at deriving practice-oriented recommendations from the interview data, drawing implications from the study findings to guide the development of support interventions. The workshop will be conducted by the project team and moderated by the principal investigator. The advisory board members, reflecting a range of backgrounds, will be divided into small groups, and each group will address a specific theme appropriate to the respective board members’ expertise. The workshop will also target the further development of the interview results gathered in study phase 2. All discussion at the advisory board workshop will be audiotaped and analyzed using thematic content analysis [61], in order to extract key points to guide the development of support interventions.

#### Phase 5: conclusions and recommendations

In study phase 5, the project team will formulate and process the final recommendations for support interventions and transfer them to an online survey using UNIPARK software (Questback GmbH, Köln, Germany). By way of a Delphi study [62] consisting of two to three rounds, the project team will seek consensus for each recommendation, with regard to relevance and feasibility. Participants in the Delphi study will include advisory board members and practitioners in the field of end-of-life care, with particular expertise in migration and mobility, as well as psychosocial care for caregivers in end-of-life contexts. The practitioners will include representatives of: (1) the German Association for Palliative Medicine (DGP), (2) the German Hospice and Palliative Care Association (DHPV), (3) Landesstützpunkt Hospizarbeit und Palliativversorgung Niedersachsen e.V., (4) Palliativstützpunkt Hannover and (5) the Lower Saxony branch of the German Association of General Practitioners. An address register will be compiled.

The Delphi study is expected to provide valuable advice for practice, since the participating practitioners will be best placed to advise on the relevance and feasibility of the preferences and needs expressed by LD caregivers in the semi-structured interviews. Specifically, participants in the Delphi study will indicate the relevance and feasibility of each recommendation on a 4-point verbal rating scale, providing additional free-text comments, if desired. All data from the Delphi study will be analyzed using descriptive statistical analysis. Feedback from the first round will be provided to all participants in the subsequent round, with the aim of increasing consensus. Recommendations with at least 80% agreement will be considered approved. All consensus recommendations will be provided as a project result.

Further to the Delphi study, the research team will assess whether core thematic dimensions can be carved out for the operationalization of a quantitative assessment instrument to measure the burden and support needs of LD family caregivers at the end of life. The project and its results are expected to serve as a basis for further evidence-generating studies.

### Study population

Study phase 2 will involve the following inclusion criteria: LD caregivers of patients ≥ 18 years old, suffering from a malignant or non-malignant life-limiting chronic disease, including HIV/AIDS (ICD-10: B20–24), a malignant neoplasm (IDC-10: C00–97), a chronic heart condition (ICD 10: I00–I52), chronic kidney disease (ICD-10: N18, N28, I12, I13), chronic liver disease (ICD-10: K70–77), chronic respiratory system disease (ICD-10: J40–47, J96, E84), a disease of the nervous system (ICD-10: G10, G12, G20, G23, G35, G71), dementia or Alzheimer disease (ICD-10: F00, F01, F03, G30) and/or senility (ICD-10: R54). LD caregivers must be ≥ 18 years old and unable to have daily face-to-face contact with the patient due to geographical distance (other place of residence) [16] and they must perceive themselves as an LD caregiver. All individuals who meet these criteria, irrespective of sex and ethnic background, may participate. LD caregivers who fulfil the inclusion criteria will be invited to participate in the individual interviews.

LD caregivers will be recruited via: [1] established contacts with general practitioners, palliative care units (incl. palliative care consultation services), hospices and specialized palliative home care teams in the Hanover region; and 2) public relations (e.g. press releases, notices sent to self-help groups). If recruitment progresses slowly in the Hanover region, outreach to further palliative care units (e.g. Hamburg), home care teams and general practitioners will be used to expand the recruitment pool. This strategy has successfully been applied in the “Dy@EoL” project [56].

The advantage of the dual recruitment strategy is that it is likely to reach both relatives of patients in early palliative care (e.g. via general practitioners providing general palliative care to patients at home) and relatives of patients in a very advanced stage of disease (e.g. via hospices). The project team will evaluate the study eligibility of all patients and their family caregivers in collaboration with the relevant multi-professional palliative care teams/general practitioners.

All participating LD caregivers will be interviewed individually. Those who are living in Germany will be offered the choice between face-to-face and telephone interviews and (in the case of face-to-face interviews) allowed to choose the interview location. Interview rooms will be available at the Institute for General Practice and Palliative Care; however, on request, project team members will visit LD caregivers at a private, primary or palliative care environment of their choosing. Interviews at neutral locations will also be facilitated. Participants will be reimbursed for any travel expenses to and from the interview location, as well as for parking costs. Members of transnational families and significant others living abroad and providing LD care can participate in the study if they consent to a telephone interview. In the event that patients receive support from more than one LD caregiver, all involved LD caregivers will be invited to participate. If a participating LD caregiver experiences significant discomfort or distress during the interview, the interview will be terminated and, if agreed by the participant, continued at a later time. All LD caregivers will provide written informed consent to participate in the project, after having received comprehensive oral and written information about the nature, content and aim of the study, and study participation.

### Inclusion, exclusion and termination criteria

All participating LD caregivers whose mother tongue is not German will be offered an interpreter. Each interpreter will sign a confidentiality agreement prior to the interview. Consent forms and project information sheets will be translated for all relevant research participants. Prior to the interview, interpreters will be extensively briefed on the research topic and a research team member will review the interview questions with them and discuss specific situations that might arise.

Exclusion criteria for LD caregivers will include: (1) cognitive impairment (e.g. dementia), (2) lack of consent to participate and (3) death of the patient prior to interview. Termination criteria will comprise: (1) significant emotional distress during the interview, (2) insufficient cognitive skills to answer the interview questions and (3) withdrawal of project participation.

### Field access and feasibility

The Institute for General Practice and Palliative Care belongs to a network of 250 academic teaching practices that contribute to research and education. Moreover, the Institute for General Practice and Palliative Care currently collaborates with regional and national palliative care units, hospices and specialized palliative home care teams for the purpose of participant recruitment to several palliative care research projects. In this study, general practitioners and members of the multi-disciplinary palliative care teams will evaluate, in close cooperation with the project team, whether a patient is receiving LDC from a family member, in order to assess potential study eligibility. Eligible LD caregivers will be approached by a project team member, who will invite them to participate in an individual face-to-face interview.

Generally, family members report benefits from participating in end-of-life care research, and show high willingness to participate [63–65]. Hence, the anticipated recruitment of 30–35 LD caregivers is feasible. Participants will be asked about their motivation to participate in the project and eligible participants who decline participation will be asked for their reasons. The participation rate and reasons for (non-)participation will be recorded and entered into IBM SPSS Statistics. If necessary, these recruitment data will be used to enhance the recruitment strategy.

### Advisory board

To gain further valuable input and to differentiate the findings from research on other groups and contexts (e.g. local caregivers to end-of-life patients), a high quality multi-faceted advisory board will support the research and project team throughout the project lifetime. Specifically, the advisory board will support the project team in: (1) reviewing the project design and methodology, (2) contributing to the development of the semi-structured interview guide and socio-demographic quantitative assessment instrument, (3) refining the recruitment strategy (incl. the involvement of interpreters), (4) reflecting on any content and methodological issues emerging from the data collection and (5) finalizing the recommendations for support interventions, in collaboration with the Delphi study. Furthermore, the advisory board will provide constructive advice on the recruitment of under-represented groups.

The board will enhance the relevance, quality and acceptance of the research project by co-shaping the project and advising on the prioritization of the results [66]. It will operate via three in-person meetings (project months 4, 8 and 21, respectively) and one Delphi study (project month 23). The board will include researchers on end-of-life care, with expertise in the research methods (e.g. qualitative interviewing of vulnerable groups, recruitment of participants with immigrant background); practitioners of direct end-of-life care involving patients and caregivers, who will be particularly called on to contribute to the third advisory board meeting and the Delphi study, on the basis of their valuable insight into the practical relevance and feasibility of the recommendations for support interventions; representatives from recruitment partners and caring professions (i.e. physicians, nurses, case managers, counsellors, social workers, psychologists, psycho-oncologists); researchers with expertise in working with interpreters to engage with more diverse participants; and patient representatives.

### Risk to subjects

This is a low-risk project, involving no interventions that are likely to cause a side effect. Due to the narrative nature of the project, participating LD caregivers may experience discomfort (e.g. anger or sadness) when discussing their loved one’s life-limiting illness/death and their own grief experience. However, studies using comparable designs have reported a low participant burden [67–69]. Upon request, resources will be provided to participants to counter any discomfort they may feel, and referrals will be made to counselling services, where appropriate. The principal investigator will be present or accessible during all interviews. It is not anticipated that suicidality will emerge within this project; however, if this should arise, the principal investigator will determine whether additional safety precautions are needed.

### Training of project team members

The project design demands project team members with high flexibility. Two interviewers will be trained prior to the data collection to foster flexibility regarding interview scheduling and empathetic and mild communication. Interviewers will be trained to observe specific reactions of stress and burden and to provide crisis interventions, where needed. The first interviews will be conducted by two interviewers, to ensure consistency, whereas subsequent interviews will be conducted by one interviewer, only. Both interviewers will receive regular supervision.

### Data handling: protection of participants’ privacy, data confidentiality and archiving

Confidentiality will be maintained through the use of identification numbers to label all audio recordings and transcripts. A document containing participant names and identification numbers will be maintained separately from the audio recordings and transcripts. There will be no link between participants’ identifying information and their identification number. Consent forms will be stored separately from the data, in a locked filing cabinet. Digital files will be encrypted and stored on password protected desktop computers. Access to participant files will be limited to the principal investigator and the project team. When the results of the research are published or discussed at conferences, no participant identities will be revealed.

### Dissemination of the research results

Once the data analysis is complete and written up, articles will be published in peer-reviewed open access journals. Furthermore, the results will be presented at (inter)national conferences in the fields of end-of-life care, health services and general practice, and disseminated via the psychosocial care section of the DGP. Only data files that are lacking personally identifiable information will be maintained after the conclusion of the study. In accordance with the APA ethics code Sect. 8.14, “Sharing Research Data for Verification” [70], the project team will disseminate the results of the research in a timely manner and will not withhold unidentifiable data from other professionals seeking to verify the authors’ conclusions. Professionals seeking to use the data set for their own research must first gain permission from both the Institute for General Practice and Palliative Care at Hannover Medical School and the author(s). The Institute for General Practice and Palliative Care is well connected to the Medical Chamber of Lower Saxony (Ärztekammer Niedersachsen), the DGP, the DGP representation of Lower Saxony, the German Association of General Practitioners representation of Lower Saxony, Landesstützpunkt Hospizarbeit und Palliativversorgung Niedersachsen e.V. and Palliativstützpunkt Hannover. All of these parties will collaborate in the public dissemination, transfer and implementation of the project results, as established through former joint projects. Educational workshops for inpatient and community palliative care services will be organized to communicate the main project results. Finally, a report on the main results will be published on the website of the Institute for General Practice and Palliative Care and communicated in relevant working groups and to practitioners.

## Discussion

It is not possible to predict whether LD caregivers will receive any direct benefits from participating in this project; however, scientific evidence suggests that respondents may obtain psychological benefits from participating in the interviews [71]. Furthermore, general participation in the end-of-life research has been shown to benefit caregivers by offering them time for reflection, acknowledging their perspectives and caring roles, and giving them a voice [67, 69]. Moreover, caregivers participating in end-of-life research report that they enjoy the prospect of both helping other caregivers in similar situations and advancing service improvements [64, 72]. In after-death situations, bereaved interviewees report that they value the space to share their experiences to a listening subject. Participants in bereavement research also report emotional release [73]. Although the bereaved individuals are likely to also experience negative emotions when discussing their loss, they tend to perceive their participation in bereavement research as helpful and positive [63, 68, 73].

In the proposed research, data from participating LD caregivers will contribute to deepening our understanding of the needs of end-of-life caregivers and the factors that hamper and support their adjustment to the LDC situation, enabling us to develop improved interventions. The results of the project will be shared with participants in the form of a letter following publication in a peer-reviewed journal. On a larger scale, participants will contribute to new models of caregiving and shed light on novel caregiving issues of central importance for contemporary society and politics.

## Supplementary Information

**Additional file 1.** Interview guide

## Data Availability

A de-identified dataset may be made available upon reasonable request of the corresponding author once the study is completed.

## Abbreviations

DFG: German Research Foundation
DGP: German Association for Palliative Medicine
DHPV: German Hospice and Palliative Care Association
LD: Long-distance
LDC: Long-distance caregiving
