# Supplementary material for: Long-distance caregiving at the end of life: a protocol for an exploratory qualitative study in Germany
Source: BMC Palliat Care. 2022 May 12;21:69. doi: 10.1186/s12904-022-00967-8 (PMC9095417; doi:10.1186/s12904-022-00967-8)
Supplement: Supplementary file 1 — Additional file 1. Interview guide [file 12904_2022_967_MOESM1_ESM.docx]

Additional Table 1: Interview guide

| **Topic** | **Sample questions** |
| --- | --- |
| General experience of end-of-life caregiving over a geographical distance | Please could you tell me what it is like for you to care for your loved one at a geographical distance? |
|  | How did you come to care for your loved one at a geographical distance? |
|  | *Additional question for caregivers from abroad:* What challenges do you face in caring for your relative since you do not both live in Germany? |
| Type of support given | What tasks do you take on or what things do you do for your relative? |
|  | Please could you describe a positive/negative situation of caring for your relative that you have experienced? What do you remember most about it and why? |
| Communication & contact | How do you stay in touch with your loved one across the distance? |
| Own health & wellbeing, burden, professional life, & financial aspects | What does the situation of caring for your relative over a geographical distance do to your own life? |
|  | What challenges but also opportunities do you experience in this situation? Please could you give me an example of each? |
| Obligations & expectations | To what extent are there commitments or expectations that you will care for your loved one over the distance? |
| Contact with other caregivers | To what extent do you share the caregiving situation with other relatives? *If applicable:* Please tell me a little bit about what this looks like. |
|  | *If applicable:* What positive/negative situations have you experienced in the process? |
| Social support & relief | To what extent do you receive support in the caregiving situation over a geographical distance? |
| Outlook | If tomorrow your friend told you his/her loved one was terminally ill and he/she lives at a geographical distance from this relative, what advice would you give your friend based on your own experience?^1^ |
|  | If hospice and palliative care staff were going to design a system of support for relatives caring for a terminally ill person over a geographical distance, what do you think would be helpful to have in that support system?^1^ |
| Closure | Is there anything further related to your caregiving situation that you would like to share? |
|  | Is there another question you think I should have asked to better understand your experience? |

Legend: ^1^Formulated according to Mazanec (19).
